# Lens-free reflective topography for high-resolution wafer inspection

**DOI:** 10.1038/s41598-024-59496-4

**Published:** 2024-05-08

**Authors:** Hojun Lee, Jangwoon Sung, Seungbeom Park, Junho Shin, Hyungjin Kim, Wookrae Kim, Myungjun Lee

**Affiliations:** Mechatronics Research, Samsung Electronics Co., Ltd., 1-1 Samsungjeonja-ro, Hwaseong-si, Gyeonggi-do 18848 Korea

**Keywords:** Imaging and sensing, Super-resolution microscopy

## Abstract

The demand for high-resolution and large-area imaging systems for non-destructive wafer inspection has grown owing to the increasing complexity and extremely fine nature of semiconductor processes. Several studies have focused on developing high-resolution imaging systems; however, they were limited by the tradeoff between image resolution and field of view. Hence, computational imaging has arisen as an alternative method to conventional optical imaging, aimed at enhancing the aforementioned parameters. This study proposes a method for improving the resolution and field of view of an image in a lens-less reflection-type system. Our method was verified by computationally restoring the final image from diffraction images measured at various illumination positions using a visible light source. We introduced speckle illumination to expand the numerical aperture of the entire system, simultaneously improving image resolution and field of view. The image reconstruction process was accelerated by employing a convolutional neural network. Using the reconstructed phase images, we implemented high-resolution topography and demonstrated its applicability in wafer surface inspection. Furthermore, we demonstrated an ideal diffraction-limited spatial resolution of 1.7 μm over a field of view of 1.8 × 1.8 mm^2^ for the topographic imaging of targets with various surface roughness. The proposed approach is suitable for applications that simultaneously require high throughput and resolution, such as wafer-wide integrated metrology, owing to its compact design, cost-effectiveness, and mechanical robustness.

## Introduction

Over the past decade, semiconductor manufacturing processes have become increasingly complex, and feature sizes have continued to shrink. As a result, it is crucial to address defects in the manufacturing process via pre-inspection of wafers in terms of time and cost. Non-destructive wafer inspection using wide field of view (FOV) and high-resolution imaging has attracted attention owing to its ability to directly measure and characterize defects^1–3^. Consequently, various imaging techniques, such as atomic force microscopy (AFM), scanning electron microscopy (SEM), and transmission electron microscopy (TEM), have been adopted. AFM provides high-resolution topographic information, enabling precise characterization of surface features and measurement of surface roughness^4,5^. However, its slow scanning speed renders it unsuitable for high-throughput wafer inspection. Additionally, AFM operates in contact mode, potentially damaging delicate samples or introducing scanning artifacts. SEM and TEM provide exceptional nanoscale imaging resolution, facilitating detailed analysis of morphology and structure^6–10^. However, they are limited to vacuum environments and require complex sample preparation, such as metal coating and thinning. These are time-consuming processes that induce surface charging and affect the original properties of the sample owing to interaction with the electron beam.

To overcome these limitations and enhance the resolution and FOV of wafer inspection, novel computational imaging techniques, such as ptychography, have emerged as promising approaches. In the field of ptychography, different approaches have been developed to address the challenges of high-resolution imaging. This includes single aperture ptychography, Fourier ptychography, and near-field ptychography. Each technique offers unique advantages, but they also come with certain limitations. Single-aperture ptychography is a versatile technique independent of specific optics; hence, it is applicable across various wavelength ranges, including extreme ultraviolet (EUV)^11–13^ and X-ray^14–17^. This feature facilitates imaging in various spectral regions and paves the way for diverse scientific and technological applications. However, single-aperture ptychography is limited by its inability to utilize the illumination spatial spectrum, which restricts the extraction of high spatial frequency information from illumination patterns. Fourier ptychography uses multiple illumination angles to expand the spatial spectral bandwidth and enhance image resolution^18–22^. It overcomes diffraction limits by capturing a set of low-resolution images and combining them using computational algorithms. However, it requires optical instruments like lenses and mirrors, which limit its application to the visible and infrared wavelength regions. In contrast, near-field ptychography incorporates a diffuser into the imaging setup, creating a speckle pattern that carry high-frequency information beyond the diffraction limit^23–29^. This technique simultaneously achieves a wide FOV and improved resolution. However, it requires the detector and sample to be in proximity, making it suitable for transmission-based configurations, particularly for the imaging of transparent biological specimens and artificial targets. Despite the exceptional hardware capabilities of near-field ptychography, its application to reflection geometries, which are essential for semiconductor device inspection because wafers are fabricated on Si substrates and reflective signals are dominant in the visible or EUV spectral regions, remains relatively unexplored.

This study proposes a reflective speckle-based lens-less imaging microscopy (Re-SLIM) method for wafer inspection, simultaneously offering high-resolution imaging and a wide FOV. From a hardware perspective, the system developed in our study shares similarities with near-field ptychography in its use of speckle illumination. However, it distinguishes itself by adapting this approach into a lens-less reflection-type detection system, making it easily applicable to wavelengths such as EUV. On the algorithmic side, we have departed from the traditional ePIE and rPIE algorithms^30^ commonly used in ptychography, opting instead for optimization methods to significantly reduce analysis time. The final image is computationally reconstructed from diffraction patterns acquired at various illumination positions using visible-light sources. Speckle illumination is introduced to expand the numerical aperture (${\text{NA}}$) of the imaging system and simultaneously enhance the resolution and FOV beyond the limitations of direct optical imaging. Furthermore, the image reconstruction process is accelerated using a convolutional neural network (CNN), improving computational efficiency^31^. In addition to image enhancement, we implement high-resolution topography using the reconstructed phase image and demonstrate its potential for the accurate and detailed characterization of wafer surfaces. This approach achieves topography imaging at a diffraction-limited resolution of 1.7 μm, with an FOV much greater than the illumination size. The advantages of this approach include compactness, cost-effectiveness, and mechanical robustness, making it suitable for high-throughput applications, including wafer-wide integrated metrology. The proposed system, being reflective and lens-less, is highly applicable in non-destructive EUV imaging, where traditional optics have limitations, resulting in a system with a small ${\text{NA}}$.

## Results

In optical imaging, the final resolution $d$ of a reconstructed image depends on the width of the synthesized bandpass filter in Fourier space, which is a function of the detection numerical aperture (${{\text{NA}}}_{{\text{det}}}$) of the imaging system and numerical aperture of the maximum angle of illumination (${{\text{NA}}}_{{\text{ill}}}$). This relationship can be expressed as

1 $$d = \frac{\lambda }{{{{\text{NA}}}_{{\text{ill}}}+{\text{NA}}}_{{\text{det}}}}$$

where $\lambda$ is the wavelength of the light source. Eq. (1) highlights that random speckle illumination, characterized by a superposition of wavevectors corresponding to different angles of incidence, is advantageous in achieving higher resolution by expanding ${{\text{NA}}}_{{\text{ill}}}$. Illuminating the sample from various angles facilitates capturing a broader range of spatial frequencies, which enhances the resolution and capability to visualize fine features that might remain imperceptible using traditional imaging methods. Consequently, optimizing the illumination and detection configurations becomes critical for maximizing spatial frequency content and improving the resolution of speckle-pattern illumination-based imaging.

## Experimental schematic of reflective speckle-based lens-less imaging microscopy

**Figure 1:**
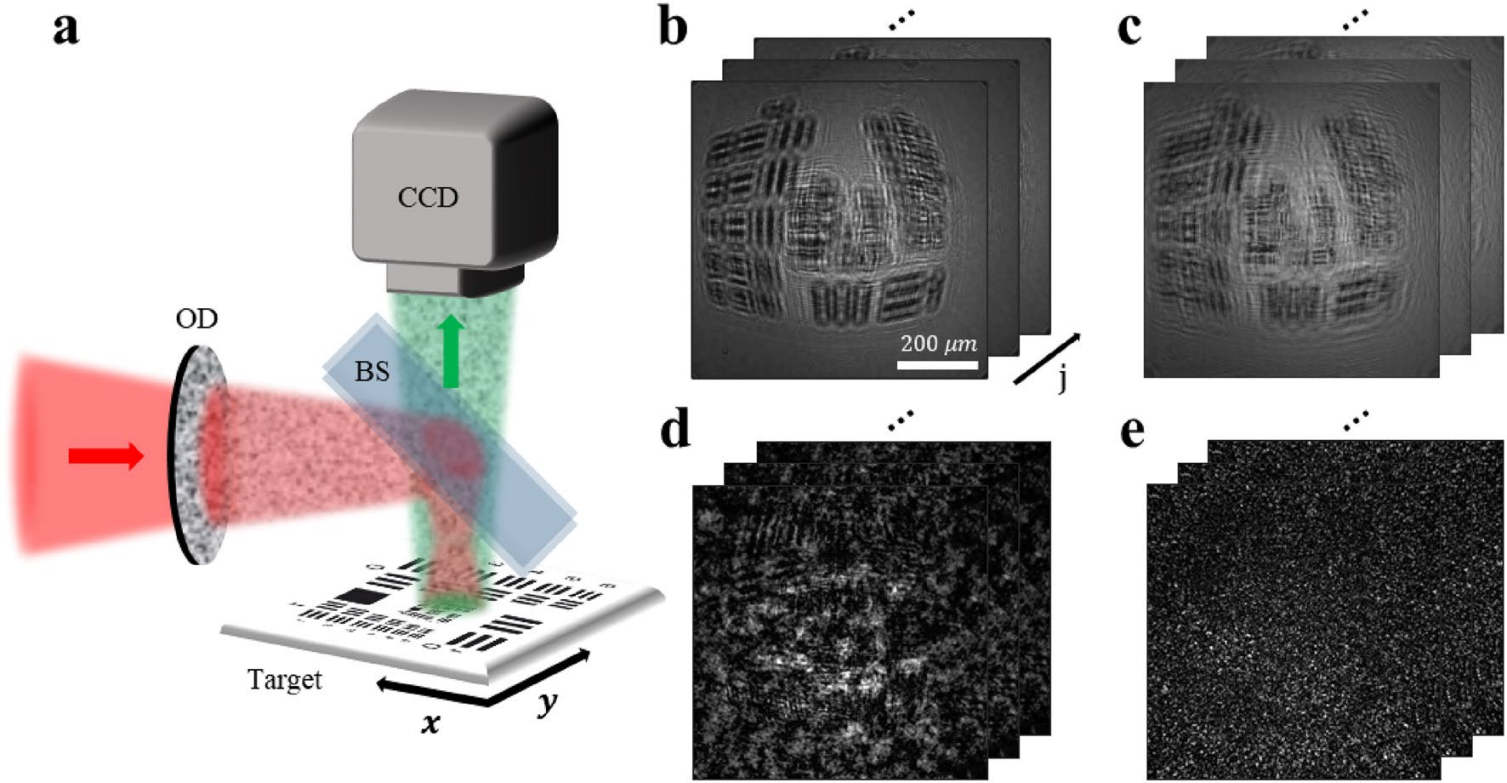
Reflective speckle-based lens-less imaging microscopy system. (**a**) Experimental schematic; the incident and reflected beams are shown in red and green, respectively, for visibility. A 405 nm laser was used as a light source, and the incidence beam was relayed by a beam splitter (BS) before focusing on the sample. Optical diffuser (OD) for speckle pattern generation. The target is loaded onto the motorized stage and moved laterally to scan the illuminated positions. (**b**) Diffracted intensity images obtained for each illuminate position ${{\varvec{r}}}_{{\text{j}}}$ in the absence of OD. Images are distorted and blurred owing to the wavefront curvature caused by the diverged illumination. (**c**) Images numerically backpropagated to the sample plane ${\varvec{r}}$. The decrease in signal-to-noise ratio renders it impossible to restore high-resolution images using only a single measured intensity image. (**d**–**e**) Measured images obtained using a weak and strong diffuser, respectively. The diffuser characteristics determine the size and randomness of the illuminated speckle grains.

Re-SLIM is built on the same backbone as that of a typical reflection-mode imaging system with a different lens-less detection scheme (Fig. 1a, Methods and Supplementary Note 1). A camera was positioned on a plane located at a distance $z$ from the target to directly capture the backscattered wave. An optical diffuser (OD) was placed in front of the beam splitter (BS) to generate speckle patterns on the sample. In the absence of a diffuser, the diverging beam illuminates the sample, and the backscattered image experiences distortion and blurring (Fig. 1b). These images are numerically backpropagated to the sample plane; however, the high-resolution images are not restored owing to the signal-to-noise ratio (${\text{SNR}}$) degradation caused by twin images. Conversely, in the presence of an OD, the incident beam interacts with the sample, resulting in a reflected intensity image with a random pattern (Fig. 1c). The weak and strong diffusers are defined based on the diffuser characteristics that determine the size of speckle grains and level of randomness. Figures 1d and 1e show the measured images obtained using the weak and strong diffusers, respectively.

Images were acquired by raster scanning a divergence or speckle beam across the sample plane using a two-axis sample stage over a given field of illumination (FOI) area. The scanning interval $\Delta s$ was determined using the spatial coherence length ${l}_{{\text{c}}}$ of the measured image. By satisfying the condition $\Delta s\le {l}_{c}$ (see Methods and Supplementary Note 2), sufficient spatial overlapping between neighboring illuminations can be achieved to increase the diffraction information required for high-resolution image restoration. As the illuminated speckle grain size decreases, the minimum scanning interval is reduced to maintain optimal measurement conditions. For each illumination point ${r}_{{\text{j}}}$ at the sample plane $r$, the intensity image $I\left(s;{r}_{{\text{j}}}\right)$ was recorded in the camera coordinate frame with the position vector $s$. Here, the detection area of the camera, called the field of detection (FOD), was sufficiently widened to capture the entire profile of the reflected wave broadened by the illumination pattern. As discussed in Methods, the final image with an FOV of 1.8 × 1.8 mm^2^ and resolution of 1.7 μm was reconstructed in a typical FOI of 100 × 100 μm^2^ for samples with various FODs. The FOV of the final image was affected by the combination of FOI and FOD; hence, the optimal sizes of FOI, FOD, and scanning interval were selected based on the FOV, resolution, and image acquisition time. As the FOI and FOD are expanded, and more fine patterns are illuminated, it is expected that the FOV and resolution of the reconstructed image will improve, but with a concomitant increase in the number of data measurement.

The proposed Re-SLIM differs from the existing reflection-based approaches in illumination method and detection configuration. In contrast to the conventional systems that use optics to deliver a beam from the sample to detector, Re-SLIM eliminates the requirement for lenses and other optical components during the detection process. This design simplicity reduces the size and complexity of the system while enhancing its durability and reliability. Re-SLIM can be advantageous in its applicability to reflective EUV imaging, particularly for photo masks or wafer inspection^11–13^, wherein the geometric structure of the system limits the ${\text{NA}}$ owing to the limitations of traditional optics. By leveraging the properties of EUV reflection geometry and harnessing the unique combination of speckle-based illumination and lens-less detection, Re-SLIM can potentially overcome the limitations of conventional methods and facilitates the application of high-resolution EUV imaging in inspection.

## Image processing and reconstruction

In optical reflectance imaging, the signal that returns to the detector must be disentangled into the probe function $\psi$ induced by the illumination system and object function $O$ obtained using the amplitude reflectance of the target. For a given illumination point ${r}_{{\text{j}}}$ on the sample plane $r$, the reflected intensity image $I\left(s;{r}_{{\text{j}}}\right)$ recorded at a position ${\varvec{s}}$ on the camera plane is expressed as follows:

2 $$I\left(s;{r}_{{\text{j}}}\right) ={\left|{H}_{{\text{z}}}\{\psi (r)O\left(r-{r}_{{\text{j}}}\right)\}\right|}^{2}$$

where ${H}_{{\text{z}}}\left\{\chi (r)\right\}={F}^{-1}[F\{\chi \left(r\right)\}{e}^{-2\pi iz/\lambda \sqrt{1-{q}^{2}{\lambda }^{2}}}]$ represents free-space propagation of a field $\chi \left(r\right)$ over a distance $z$ at a detector position, and $F$ denotes the Fourier transform operation with a reciprocal coordinate $q$. Herein, multiple measurements with spatial overlap are required to exploit the convolution relationship between $\psi$ and $O$. The overlapped measurements ensure that each part of the object is illuminated by multiple patterns, thereby recovering the complete electric field information of the object and probe without prior knowledge. This approach utilizes the translational invariance of illumination and extracts high-resolution object features.

**Figure 2:**
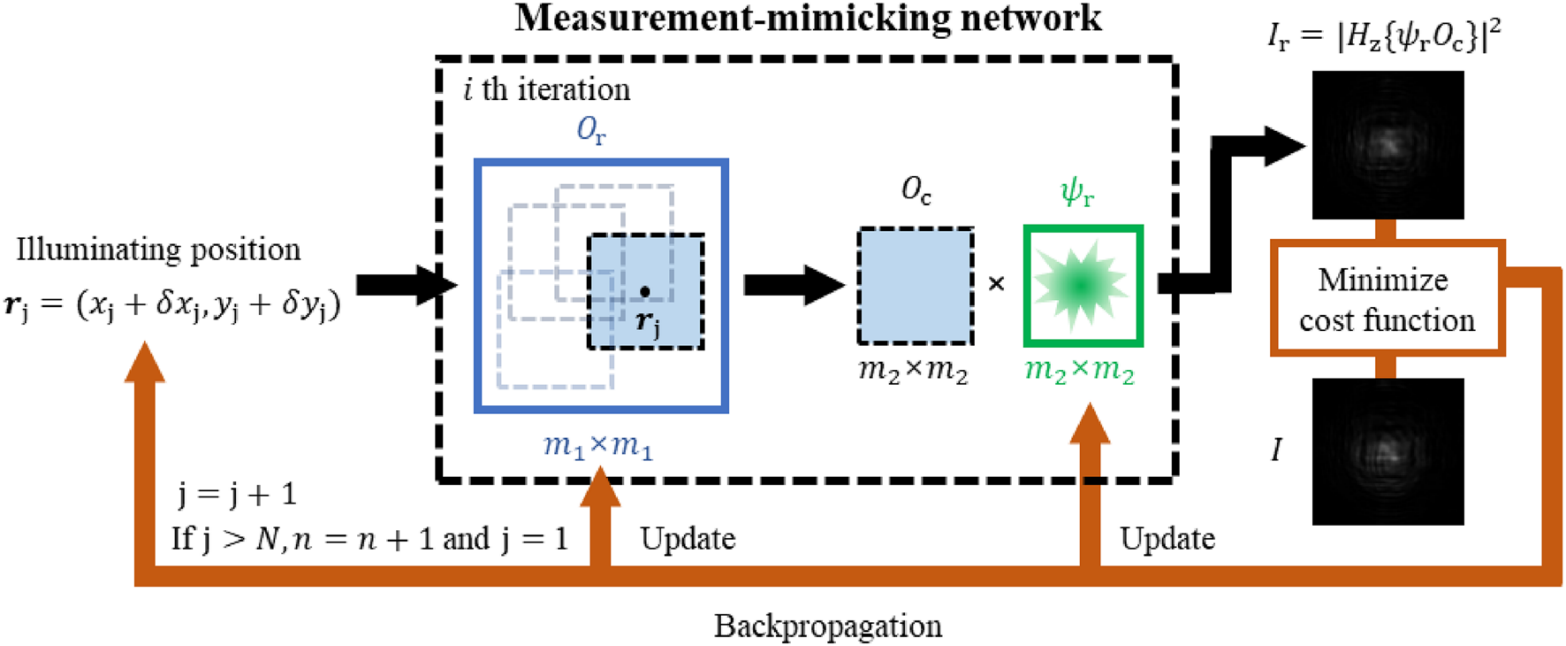
Flow chart of the image reconstruction algorithm. The network input corresponds to the ${\text{j}}$-th measured diffractive image $I$ and illuminated position ${{\varvec{r}}}_{{\text{j}}}=({x}_{{\text{j}}}+\delta {x}_{{\text{j}}},{y}_{{\text{j}}}+\delta {y}_{{\text{j}}})$, where $(\delta {x}_{{\text{j}}}$, $\delta {y}_{{\text{j}}})$ represents the measurement error in the scanning position. The reconstructed complex functions ${O}_{{\text{r}}}$ and ${\psi }_{{\text{r}}}$ are considered as the two-channel learnable filter of the convolutional layer. The number of pixels in ${\psi }_{{\text{r}}} ({m}_{2}\times {m}_{2})$ is determined using FOD, whereas that in ${O}_{{\text{r}}}$ (${m}_{1}\times {m}_{1})$ is determined using both FOD and FOI. Therefore, a new cropped object function ${O}_{{\text{c}}}$ is defined by selecting ${m}_{2}\times {m}_{2}$ pixels from the illuminated position. Using zero-padding, the pixel number can be adjusted based on the reconstruction time and FOV of the reconstructed image. The output of the network ${I}_{{\text{r}}}$, propagated to the detector plane $s$ using the element product of ${\psi }_{{\text{r}}}$ and ${O}_{{\text{c}}}$, is compared with the $I$. A single iteration is completed after the same process is performed for all $N$ measured images. The optimization process of the network iterates $i$ times until Eq. (3) is minimized.

To perform the deconvolution of $O$ and $\psi$, the image reconstruction algorithm^31^ was applied to the measured dataset. Figure 2 shows the proposed CNN model, which consists of an input layer that accepts the ${\text{j}}$-th measured image $I\left(s;{r}_{{\text{j}}}\right)$, output layer that evaluates the diffractive image , and convolution layers with two channels for the real and imaginary parts of the reconstructed object ${O}_{{\text{r}}}$ and probe ${\psi }_{r}$ functions, respectively. The pixel numbers of ${O}_{{\text{r}}}$ and ${\psi }_{{\text{r}}}$ are influenced by the FOD and FOI; however, they can be adjusted via zero-padding for versatile changes. Increasing the pixel number of ${O}_{{\text{r}}}$ or ${\psi }_{{\text{r}}}$ can result in a broader FOV depending on illumination characteristics; however, it may yield longer image reconstruction times (Supplementary Note 3). During the training process, the two-channel functions were recovered using the measured diffraction images. This light propagation model mimicking the neural network predicts the image ${I}_{{\text{r}}}={\left|{H}_{{\text{z}}}\{{\psi }_{{\text{r}}}{O}_{{\text{c}}}\}\right|}^{2}$ propagated forward by a distance of $z$ using the product of ${\psi }_{{\text{r}}}$ and the cropped object function ${O}_{{\text{c}}}$. In the backward pass, the difference between ${I}_{{\text{r}}}$ and $I$ is back-propagated to the convolution layer and the two-channel functions are updated to ${O}_{{\text{r}}}{\prime}$ and ${\psi }_{{\text{r}}}{\prime}$. Therefore, the training process of the proposed model is analogous to a minimization process for the following loss function:

3 $${O}_{{\text{r}}}{\prime}, {\psi }_{{\text{r}}}{\prime}=\underset{{O}_{{\text{r}}}, {\psi }_{{\text{r}}}}{{\text{argmin}}}\{{\Vert {I}_{{\text{r}}}-I\Vert }_{1}+{\eta \| \nabla {O}_{{\text{r}}}\| }_{1}\}.$$

Here the L1-norm is applied to intensity image to measure the difference between the predicted and actual measurements. The second term of Eq. (3) represents the total variation (TV) regularization, wherein the empirically determined coefficient $\eta$ controls the regularization strengths^32^ (Supplementary Note 4). While this process was repeated $i$ times, the neural network model was trained using the Adam optimizer until the fidelity between the measured and reconstructed images decreased below a certain threshold (Supplementary Video 1). Consequently, the convergence time of ${O}_{{\text{r}}}$ and ${\psi }_{{\text{r}}}$ was reduced by 83% compared to the traditional image reconstruction algorithms^30^ (Supplementary Note 5). Details on the image reconstruction algorithm are available in Supplementary Note 6.

The proposed model is advantageous in its ability to adjust the number of learnable variables by increasing the number of convolutional layers. For instance, it corrects the position measurement errors $(\delta {x}_{{\text{j}}}$, $\delta {y}_{{\text{j}}})$ caused by the scanning stage backlash or other noise factors that occur experimentally. Blurred artifacts can appear in the final restored image when the scanning position information in the input layer is inaccurate. To address this, a position error term is introduced as a trainable variable in the network model. By iteratively adjusting this term, the model minimizes the discrepancy between the captured (${I}_{{\text{j}}}$) and desired ($I$) images, facilitating efficient error correction. This adaptive approach ensures optimal error compensation, resulting in improved image quality and fidelity (Supplementary Note 7). This versatility allows for a broader range of applications and expands the utility of the model.

## Proof-of-concept experiment demonstrating the speckle illumination effect

**Figure 3:**
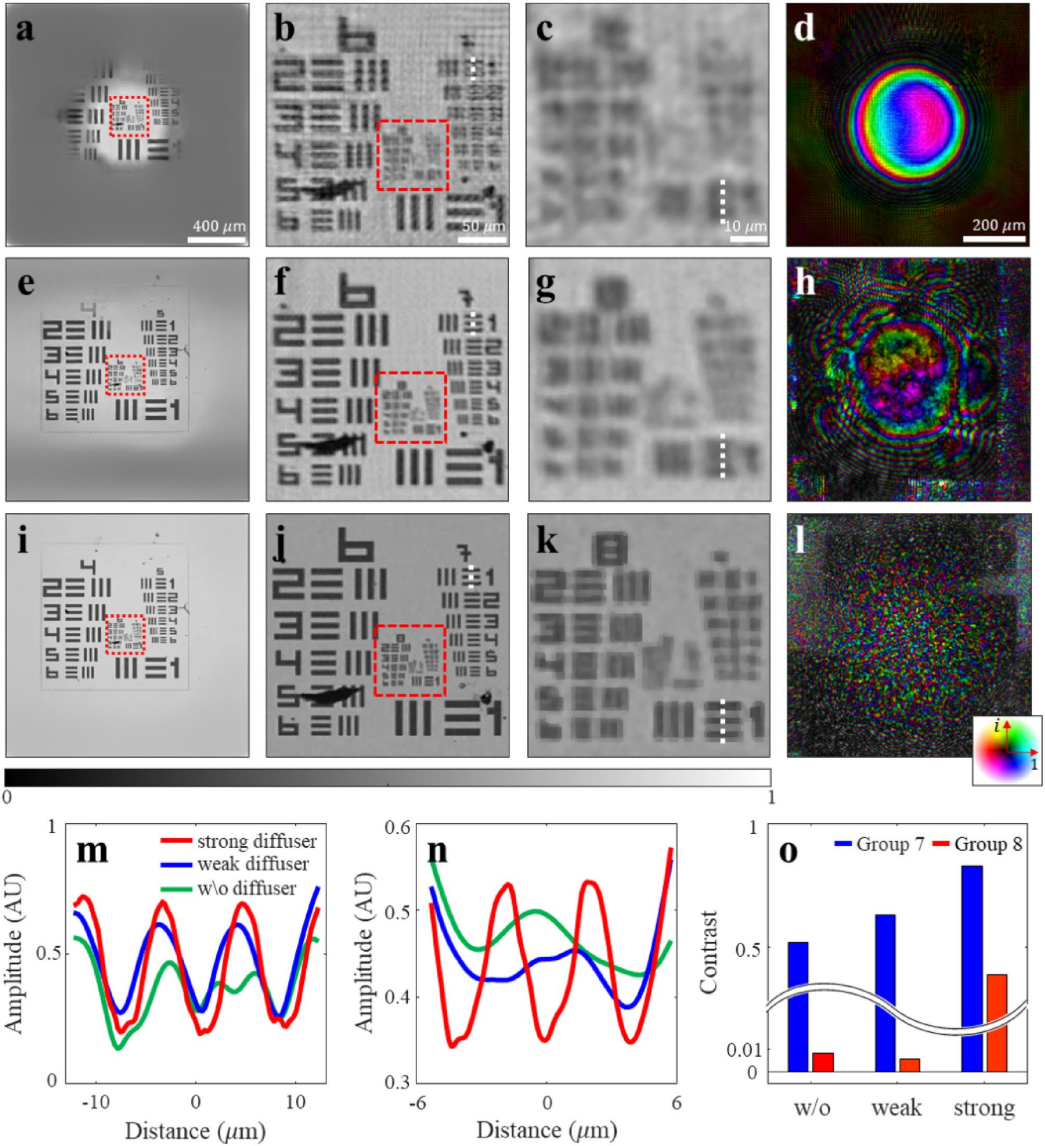
Image reconstruction fidelity based on the illumination pattern. (**a**–**d**) Reconstructed image ${O}_{{\text{r}}}$ and probe function ${\psi }_{{\text{r}}}$, respectively, without a diffuser. (**b**) and (**c**) show the magnified images of the regions within the red boxes in (**a**) and (**b**), respectively. (**e**–**h**) Reconstructed image and probe function using a weak diffuser. (**i**–**l**). The same as (**e**–**h**), but with a strong diffuser. The array sequence for all images follows as (**a**–**d**). All images are normalized for comparison. As the illumination pattern becomes more complex (**d**, **h**, and **l**), the FOVs of the images expand (**a**, **e**, and **i**) and their resolutions improve (**c**, **g**, and **k**). (**m**). Line plot graph of element 1 in group 7 of images (**b**), (**f**), and (**j**) (denoted by the white dotted line). (**n**) Line plot graph of element 1 in group 8 of images (**c**), (**g**), and (**k**) (denoted by the white dotted line). (**o**). Graph comparing the contrast of line plots for (**m**) and (**n**). For group 8 patterns, a significant contrast value of 0.4 is observed when a strong diffuser was used.

Figure 3 shows the computational reconstruction results. To demonstrate the performance of the Re-SLIM system, we compared the restored images of a USAF resolution test target under the following three conditions: without a diffuser, with a weak diffuser, and with a strong diffuser. In all cases, the image and probe function were reconstructed successfully; however, differences were observed in the image quality and shape of the probe pattern. Considering throughput, the FOV is constrained by the size of the illuminating beam in beam-scanning imaging. Therefore, the final image obtained using the diffraction patterns without the diffuser exhibited a narrower FOV of 0.8 × 0.8 mm^2^. In contrast, when a diffuser was employed, the illuminating beam was broaden such that the FOV expanded to 1.8 × 1.8 mm^2^ for a strong diffuser, requiring 4325 × 4325 pixels to represent the image. Based on the magnified images and line plot graphs for resolution comparison, the conventionally restored image was severely blurred, making it difficult to identify group 7 elements. The presence of significant background noise further deteriorated the ${\text{SNR}}$ of the image. On the contrary, the elements of groups 7 and 8 were successfully restored when a strong diffuser was used. The contrast can be used as a quantification criterion to compare the image quality. It is expressed as $\frac{{I}_{{\text{sig}}}-{I}_{{\text{back}}}}{{I}_{{\text{sig}}}+{I}_{{\text{back}}}}$, where ${I}_{{\text{sig}}}$ and ${I}_{{\text{back}}}$ represent the target and background intensity values, respectively. The contrast value of element 1 in group 8 was significantly improved using a strong diffuser.

Resolution enhancement can also be observed by comparing the image spectrum distribution ${\widetilde{O}}_{{\text{r}}}\left(q\right)=F\left\{{O}_{{\text{r}}}(r)\right\}$ in the spatial frequency domain $q$. The spatial frequency of the restored object exhibited a broader spectral bandwidth ${f}_{w}$ using speckle illumination (Supplementary Note 8). This can be attributed to the overall increase in the ${\text{NA}}$ of the system based on the Fourier relationship, indicating the presence of more detailed information regarding finer features in the reconstructed image. In each case, the ultimate achievable resolution of the final restored image was analytically estimated using ${\text{NA}}$. As described in Methods, ${{\text{NA}}}_{{\text{det}}}$, represented by the geometric design of the system, has a constant value of 0.14. However, ${{\text{NA}}}_{{\text{ill}}}$, represented by the characteristics of the employed diffuser, varies in the three cases and exhibits the corresponding values of 0, 0.04, and 0.10. By substituting the calculated ${{\text{NA}}}_{{\text{det}}}$ and ${{\text{NA}}}_{{\text{ill}}}$ values in Eq. (1), the estimated diffraction limits for the three cases were respectively 2.8, 2.2, and 1.7 μm. Experimentally, the minimum resolvable pattern size corresponded to the expected value in each case.

## Quantitative analysis of image recovery performance based on ${\mathbf{N}\mathbf{A}}_{\mathbf{i}\mathbf{l}\mathbf{l}}$

**Figure 4:**
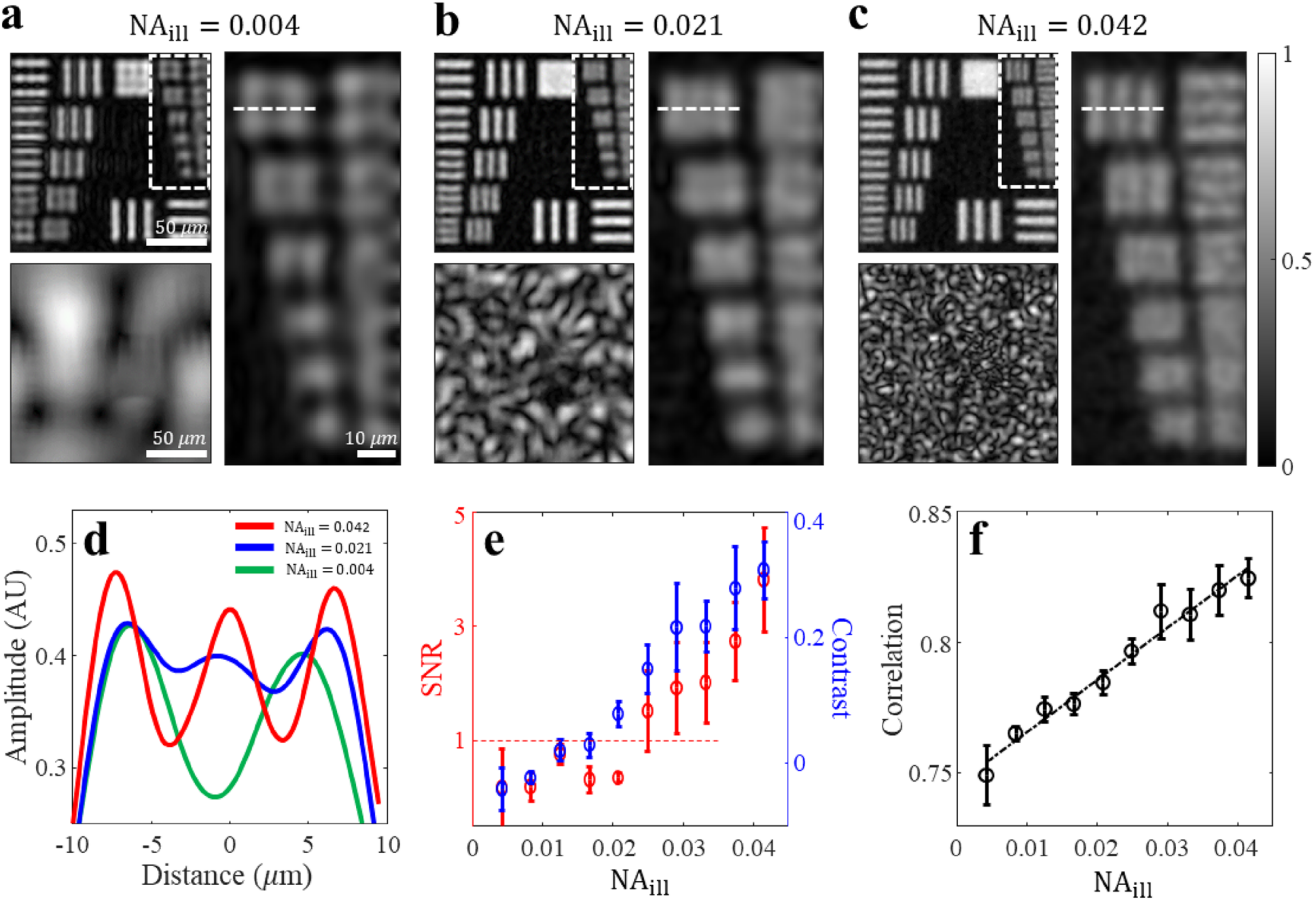
Systematic image analysis based on ${{\text{NA}}}_{{\text{ill}}}$. (**a**–**c**) Restoration image (top left) and illumination pattern (bottom left) for each case with ${{\text{NA}}}_{{\text{ill}}}$ values of 0.004, 0.021, and 0.042. The magnified region indicated by the white dotted box is shown on the right-hand side. (**d**) Line plot graph denoted by white dotted lines in (**a**–**c**). (**e**) ${\text{SNR}}$ (red) and contrast (blue) of the pattern in (**d**) as a function of ${{\text{NA}}}_{{\text{ill}}}$. An ${\text{SNR}}$ value of 1 indicates a threshold which the pattern cannot be distinguished accurately. (**f**). Correlation between the ground truth and restored images as a function of ${{\text{NA}}}_{{\text{ill}}}$. The dotted black line represents the linear plot of data points. Based on the theoretical investigations in Methods, the physical quantities reflecting image quality exhibit a linear relationship with ${{\text{NA}}}_{{\text{ill}}}$. In (**e**) and (**f**), the analysis was performed by statistically averaging the results using five different random patterns while keeping the same ${{\text{NA}}}_{{\text{ill}}}$ value for each data point.

We evaluated the image recovery performance based on ${{\text{NA}}}_{{\text{ill}}}$ and performed quantitative image analysis using simulations. In Figs. 4a–c, the top-left images represent the restored amplitude images of the object, and the bottom-left images correspond to the speckle patterns of illumination with ${{\text{NA}}}_{{\text{ill}}}$ values of 0.004, 0.021, and 0.042. As ${{\text{NA}}}_{{\text{ill}}}$ increased, the incident beam contained more high-angle components, resulting in smaller and more complex speckle patterns. At ${{\text{NA}}}_{{\text{ill}}}=0.004$, the magnified images on the right-hand side and line profiles along the white dotted lines (Fig. 4d) were unable to differentiate the three bars. However, at ${{\text{NA}}}_{{\text{ill}}}=0.042$, the bars were clearly differentiated. Therefore, employing more diffusive illumination patterns increases the cutoff frequency bandwidth of the system, facilitating the restoration of smaller features in the object.

In imaging, ${\text{SNR}}$ is a crucial quantitative assessment parameter, which is determined by the ratio of the target image intensity ${I}_{{\text{sig}}}$ to the standard deviation of random background noise ${\upsigma }_{{\text{back}}}=std({I}_{{\text{back}}})$. The ${\text{SNR}}$ was estimated using the relationship between the resolution limit and ${{\text{NA}}}_{{\text{ill}}}$, which is expressed by Eq. (1). Examining both sides of the Fourier transformation and reciprocal space reveals that the spatial frequency bandwidth ${f}_{w}$ of the object is directly proportional to ${{\text{NA}}}_{{\text{ill}}}$. Consequently, the increase in ${{\text{NA}}}_{{\text{ill}}}$ expands the number of orthogonal modes in the spatial spectrum. Hence, the ${\text{SNR}}$ can be estimated as follows (see details in Methods and Supplementary Note 8):

4 $${\text{SNR}}=\frac{{I}_{{\text{sig}}}}{{\upsigma }_{{\text{back}}}}={k{\text{NA}}}_{{\text{ill}}}$$

where $k$ is a proportionality constant. For statistical verification, we selected five random speckle patterns corresponding to each ${{\text{NA}}}_{{\text{ill}}}$ and generated a set of diffraction intensity images. The final images were reconstructed from these image sets, and the ${\text{SNR}}$ was plotted as a function of ${{\text{NA}}}_{{\text{ill}}}$ as shown in Fig. 4e. ${\text{SNR}}$ and ${{\text{NA}}}_{{\text{ill}}}$ exhibit a well-fitted relationship, wherein an ${\text{SNR}}$ value less than 1 indicates the inability to resolve the corresponding pattern. The contrast, which is another quantitative measure reflecting the image quality, followed a similar trend as the ${\text{SNR}}$, indicating a consistent relationship between the two metrics. We further investigated the correlation between the reconstructed and ground truth images and observed a strong correlation that aligned well with ${{\text{NA}}}_{{\text{ill}}}$, as shown in Fig. 4f. These results confirmed the validity of our analysis.

## High-throughput and high-resolution topography for wafer inspection

Re-SLIM is advantageous in its ability to computationally reconstruct the full electrical field information of an object in a reflection configuration. The retrieval of the object phase has various applications, including the prediction of object properties^12^ and measurement of surface roughness^18,25^. Herein, accurate topography measurements are essential to ensure the quality and performance of semiconductor devices. However, achieving high throughput and accurate topography remains challenging owing to the tradeoff between the FOV and spatial resolution. This section experimentally demonstrates the performance of Re-SLIM on various reflective samples with different patterned structures. Additionally, the proposed method was successfully applied to a scratched wafer model, demonstrating its utility in wafer inspection applications. By implementing speckle illumination, we can accurately capture the detailed surface information and expand the FOV, thereby achieving high-throughput precise characterization.

**Figure 5:**
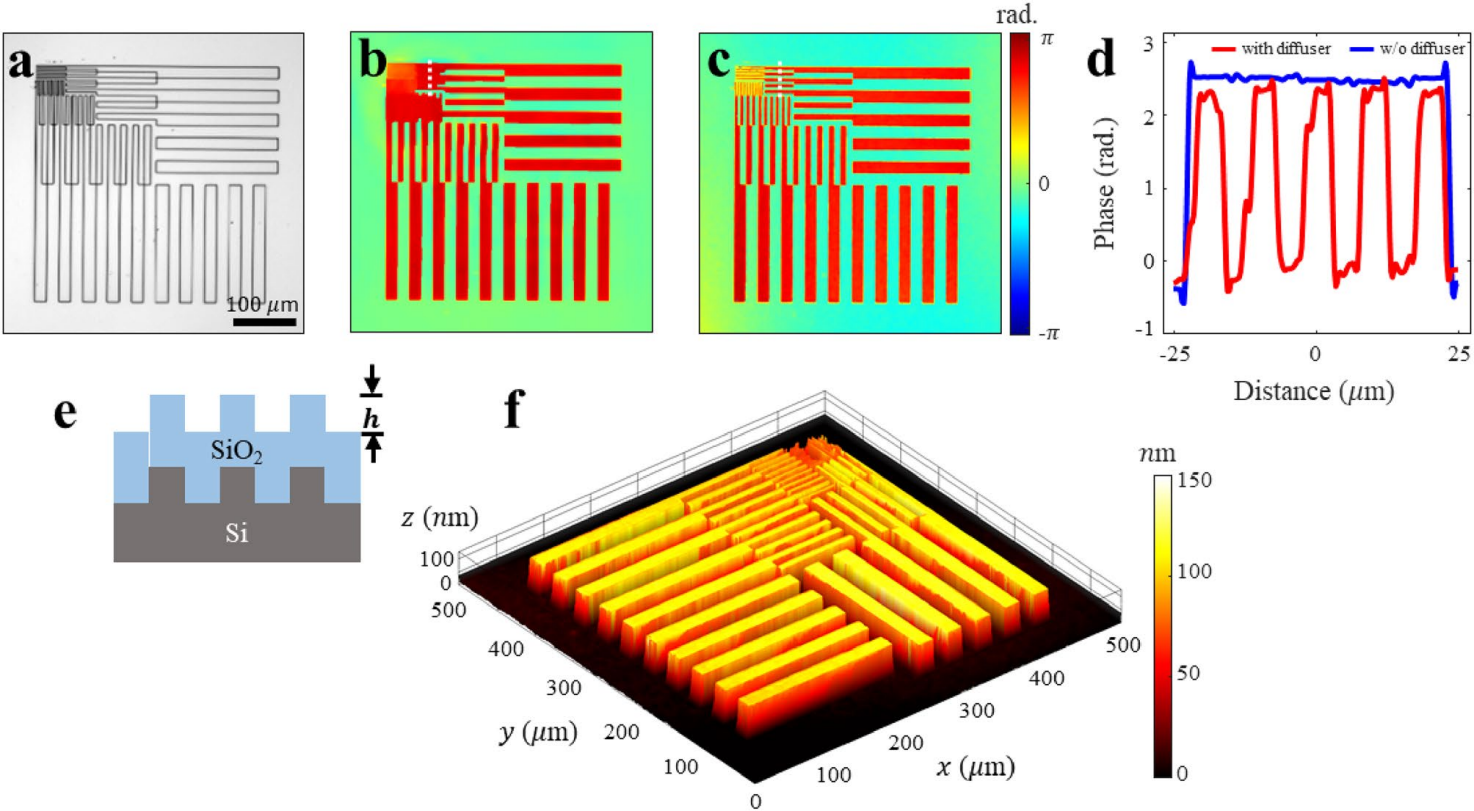
Surface height evaluation using a high-resolution phase image. (**a**) Optical microscope image of the home-made resolution test target, exhibiting patterns with bar spacing values in the range 19 − 2.4 μm. (**b**) and (**c**) Reconstructed phase images in the absence and presence of speckle illumination, respectively. (**d**) Line plot graphs of the positions indicated by the white dotted lines in (**b**) and (**c**). (**e**) Cross sectional layout of the prepared resolution test target. A line-space structure with a height $h$ of 100 nm is etched and a layer of SiO_2_ with a thickness of $2h$ is deposited on an Si substrate. (**f**) 3D surface plot reconstructed using the high-resolution phase image of **c**. For the structure with a spacing of 4.8 μm, the obtained height values are in 92.5% agreement with the reference values.

The home-made resolution test target, simulating the surface roughness encountered during the wafer fabrication processes, was used as the sample (Fig. 5a). A grating structure with a height of 100 nm was etched on an Si substrate, and an SiO_2_ layer of the same height was deposited on the top (Fig. 5e). This model encompasses structures with varying half-pitches ranging from 19 μm for the largest structure to 2.4 μm for the smallest structure, enabling the quantitative evaluation of the resolution. Figures 5b and c represent the final restored phase images obtained using conventional divergence-wave and speckle illuminations, respectively. In both cases, large structures were effectively resolved. However, structures with a size of 4.8 μm were not identifiable as shown in Fig. 5b, whereas structures as small as 2.4 μm were resolved as shown in Fig. 5c. This is further validated by the line plot in Fig. 5d. Because the structures of the Si and SiO_2_ layers were identical, the phase contrast was determined based on the difference in the optical path length caused by the variation in the height of the air medium. Therefore, the surface height $h\left(r\right)$ of the sample was calculated using the reconstructed high-resolution phase image $\Phi \left(r\right)=arg({O}_{{\text{r}}}\left(r\right))$ as follows:

5 $$h\left(r\right)= \frac{\lambda }{4\pi }\Phi \left(r\right).$$

The calculated results are presented in the topology diagram shown in Fig. 5f. As shown in Fig. 5d, the height of 107 nm was in accordance with the value measured (116 nm) using the white light interferometry (WLI) reference tool with a 92.5% agreement.

**Figure 6:**
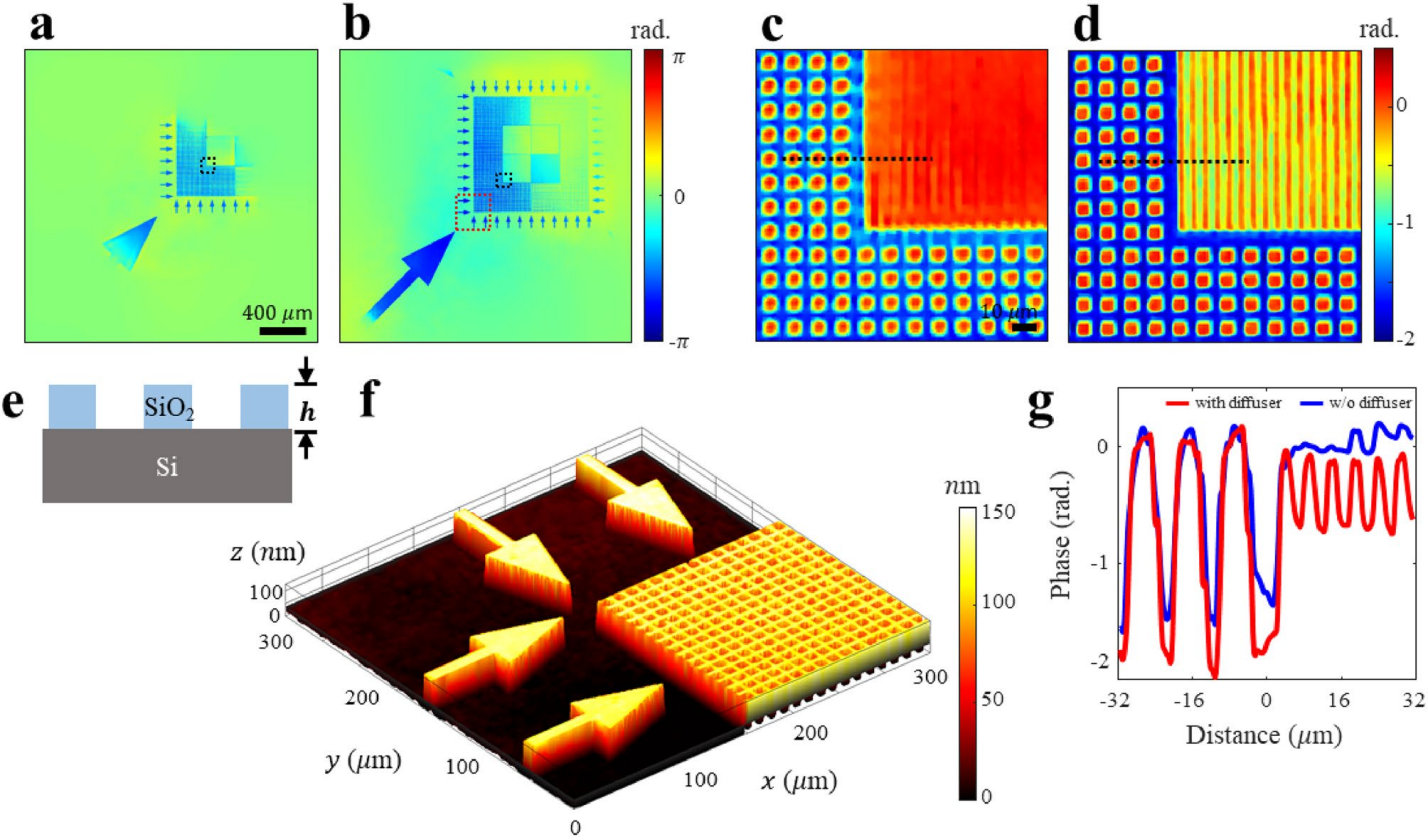
Implementing high-resolution topography on the AFM test target. (**a**) and (**b**) Reconstructed phase images in the absence and presence of speckle illumination, respectively. (**a**) has a narrow FOV of 0.8 × 0.8 mm^2^, whereas (**b**) has a wide FOV of 1.8 × 1.8 mm^2^. The magnified images of the regions indicated by the black boxes in (**a**) and (**b**) are shown in (**c**) and (**d**), respectively. (**e**) Cross sectional layout of the AFM test target. A regular pattern of SiO_2_ is fabricated on a flat Si substrate. The height $h$ of the designed SiO_2_ is 100 nm. (**f**) 3D surface plot of the region indicated by the red box in (**b**). (**g**) Line plot graph of the positions indicated by the black dotted lines in (**c**) and (**d**) Evidently, the line-space patterns with a spacing of 2.5 μm are resolved when a diffuser was presented.

We also conducted experiments with an AFM test target (HS-100MG). This target represents a common structure resembling the frequent forms observed during the wafer fabrication processes, wherein SiO_2_ with a thickness of 100 nm is manufactured on the surface of an Si substrate at regular intervals (Fig. 6e). As observed in the magnified image, the target consists of cylindrical wells and grating structures with a half-pitch of 5 and 2.5 μm, respectively. While examining the restored phase image of the FOV, the speckle illumination extended the observable FOV from 0.8 × 0.8 to 1.8 × 1.8 mm^2^, represented by the image composed of 3404 × 3404 pixels (Figs. 6a and b). Additionally, the resolution was enhanced owing to the increased ${\text{NA}}$ resulting from speckle illumination (Figs. 6c,d). Figure 6g shows that the phase image reconstructed using the OD resolves the fine line-space structure. This sample exhibited beam reflection at the Si and SiO_2_ surfaces. However, because the signal from the Si surface is dominant, the reflected signal from the SiO_2_ surface can be disregarded. Based on the sample composition, the difference in the optical path length arises from the height and refractive index $n$ of SiO_2_. Therefore, the surface height $h\left(r\right)$ can be determined as follows:

6 $$h\left(r\right)=\frac{\lambda }{4\pi \left(n-1\right)}\Phi \left(r\right).$$

As shown in Fig. 6f, the 3D structure was successfully restored from the reconstructed phase image. Using speckle illumination, the matching rate of the reconstructed height to the nominal value increased from 76.9 to 93.5%.

**Figure 7:**
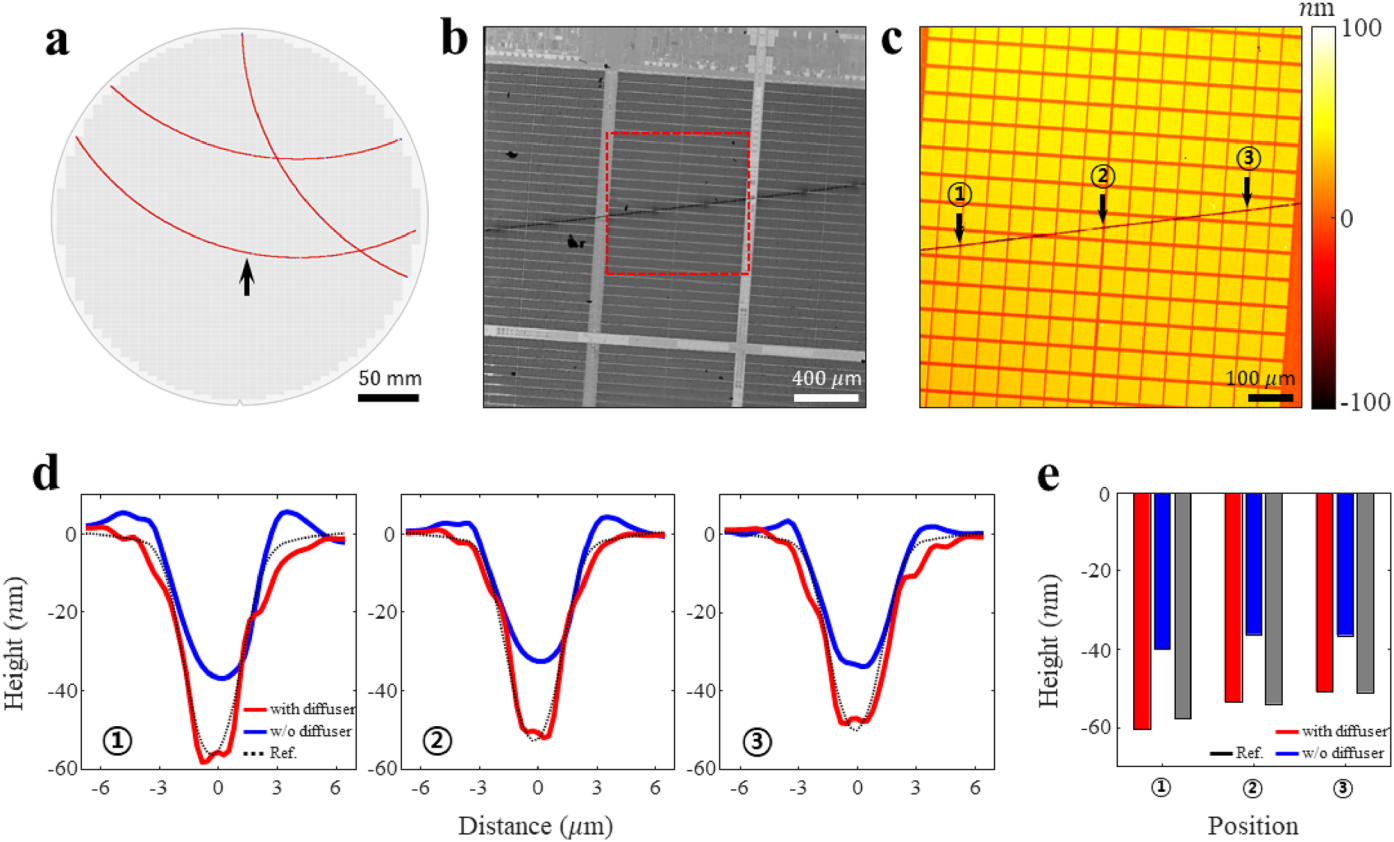
High-resolution topography for measuring the scratch depth. (**a**) Scratch model on the wafer surface. This is a model of the scratch defect generated during the wafer manufacturing process, particularly the chemical-mechanical polishing (CMP). (**b**) Reconstructed amplitude image of the position pointed by the arrow in (**a**). (**c**) High-resolution topography of the scratched area indicated by the red box in (**b**). (**d**) Line plot graph of scratch depths corresponding to each position in (**c**). The red and blue lines are with and without the diffuser, respectively. The black line is the reference value of the scratch depth. (**e**) Graph of scratch depth quantification with position. A comparison of reference values reveals that the presence of speckle illumination improved the accuracy of scratch depth measurement from 69.3 to 98.1% compared to its absence.

The proposed approach can be applied to all the steps of wafer area inspection, which simultaneously require a high resolution and large FOV. Scratches are common defects introduced during the wafer fabrication process. However, there is no available tool that quantitatively characterizes the location and depth of scratches with high throughput. Additionally, accurate measurement of scratch depths requires high-resolution images because the width of a scratch narrows as its depth increases. We applied high-resolution topography to the wafer scratch sample to quantitatively characterize the location and depth of scratches over a wide region. Figures 7b and c show the reconstructed image and the corresponding topography of the wafer scratch rendered during the chemical-mechanical polishing (CMP) process (Fig. 7a). To verify the effect of speckle illumination, the scratch cross-sections were captured at various positions with and without the diffuser (Fig. 7d). The measured surface area of the scratch was 7.2 μm, whereas the width at the deepest point was 1.4 μm. Speckle illumination can only resolve a scratch to its maximum depth. The black dashed curves indicate the scratch depth measured using the WLI reference tool. Based on the results, the scratch depths are quantified in Fig. 7e. The accuracy of scratch depth measurement in the presence of speckle illumination improved from 69.3 to 98.1% compared to its absence.

## Discussion

The Re-SLIM system represents a novel approach for high-throughput and high-resolution imaging. Its advantages are attributed to the following two components: an illumination unit that introduces speckle patterns and a lens-less detection unit that measures the reflected signals. In the illumination unit, an OD is strategically positioned to create speckle patterns on the sample. This enhances the overall ${\text{NA}}$ of the system by expanding the spatial frequency spectrum of the incident beam, consequently improving spatial resolution and ${\text{SNR}}$ s in the resulting images. Furthermore, the diffusive nature of the incident beam broadens the illuminated area, thereby boosting throughput by extending the FOVs of images. Selecting an appropriate diffuser is crucial because its characteristics dictate the resolution, FOV, and image acquisition time of the system. In contrast to the conventional reflection-based imaging systems within the detection unit, Re-SLIM eliminates the requirement of lenses and other optical components during the detection process, resulting in a more compact and robust system.

Image reconstruction in Re-SLIM involves the deconvolution of the probe function generated by the illumination system and the object function derived from the reflectance of the target. A neural network model with convolution layers is employed for image reconstruction. This model predicts the image and probe functions based on the measured diffraction images. The loss function, which quantifies the disparity between the predicted and actual measurements, is minimized during reconstruction. Consequently, the proposed model significantly enhances the speed of the imaging process, achieving a maximum reduction of 83% when compared to conventional ptychographic methods. To explore its potential applicability in wafer inspection, Re-SLIM was empirically tested on various reflective samples, including a resolution test target and wafer scratch model. The system successfully reconstructed high-resolution phase images and allowed for accurate topography measurements, even for structures with submicrometer heights and depths. The speckle illumination in Re-SLIM expands the FOV (from 0.8 × 0.8 to 1.8 × 1.8 mm^2^) and enhances the resolution (from 2.8 to 1.7 μm), providing a high-throughput and precise characterization capability.

Nevertheless, several factors will need to be considered to apply this technology for wafer inspection at EUV wavelengths in-fab. Using finer illumination patterns can enhance the resolution and FOV; however, it may cause energy loss per unit area because of diffraction. While this is not a concern when the light source output is sufficient, coherent EUV sources from high-harmonic generation (HHG) used in in-fab setups typically have outputs in the order of several hundred nanowatts. The power attenuation caused by the diffuser requires longer detector exposure times, potentially affecting the inspection throughput. Similarly, we should consider the efficient designing of EUV diffusers for these purposes. Recent advancements in the outputs of EUV source-driven HHGs^12,33–35^ and research on highly efficient reflection or transmission-type EUV diffusers^36^ make the adaptation of our proposed technique for in-fab inspection feasible.

In addition, there are considerations to be taken into account regarding the application of Re-SLIM in the visible light wavelength range. The roughness of the diffuser and the curvature of the probe light are critical factors influencing speckle imaging and its practical applications. The roughness of the diffuser affects the uniformity of speckle pattern distribution, while the curvature of the probe light determines the spatial distribution of speckles on the illuminated surface. The selection of a diffuser depends on specific application requirements, such as desired spatial resolution and sensitivity to surface roughness variations. Further research^37,38^ could focus on exploring advanced diffuser materials with controlled roughness profiles and optimizing the curvature of the probe light to enhance the performance of speckle imaging systems across various applications.

This design simplicity of Re-SLIM reduces the size and complexity of the system and enhances its durability and reliability. Based on the characteristics of the illumination and detection units, Re-SLIM is advantageous in its applicability to reflective EUV imaging, wherein the overall ${\text{NA}}$ is limited by the geometrical structure of the system owing to the limitations of traditional optics. In Re-SLIM, the absence of optics and ${\text{NA}}$ extension achieved through speckle illumination can address the limitations of EUV imaging, rendering it applicable in the semiconductor industry, particularly in photo masks or wafer inspection.

## Materials and methods

Experimental setup in Figure 1a: A 405 nm laser (OBIS, Coherent Inc.) was used as a light source, and the beam was relayed by a BS before focusing on the sample. An OD was placed behind the BS in the beam path to introduce speckle illumination into the sample. Scotch tape (cat. 104A, 3M) and vinyl chloride (PVC) were used as the strong and weak diffusers, respectively. The sample was mounted on a high-precision electric translation stage (LNR502E, Thorlabs), which facilitated the transverse movement relative to the beam. In the experiments, the Re-SLIM system was set up with a FOI of 100 × 100 μm^2^, whereas the FOD varied for each sample (see Supplementary Note 1). The 121 diffracted beams were sequentially captured using a CCD (s-CMOS, Pco. edge 4.2, 2048 $\times$ 2048 pixels, pixel size: 6.5 μm) while the illumination position was raster scanned with a step size of 10 μm. The scan parameters could be adjusted based on the diffuser type, desired resolution limit, and image acquisition duration.

Determination of ${{\text{NA}}}_{{\text{det}}}$: The experiment was performed by focusing the incident beam before it reached the sample, allowing a divergent wave to diffract from the sample and form an interference pattern on the camera plane. Supplementary Figure 1b shows a schematic of the setup in the transmission configuration. A single pixel size of the camera is denoted as $\Delta x$ and the sensor consists of $m\times m$ pixels, resulting in a total sensor size of $D=m\times \Delta x$. However, the divergent illumination beam magnified the sample plane on the detector plane by a factor of $M$. Consequently, the effective pixel size $\Delta {x}_{{\text{eff}}}$ and sample-detector distance ${z}_{{\text{eff}}}$ were determined using the geometric propagation of the illumination beam. By employing the angular spectrum method for propagation, $\Delta {x}_{{\text{eff}}}$ in the sample plane can be derived using the geometric magnification factor $M$,

7 $$\Delta {x}_{{\text{eff}}}= \frac{\Delta x}{M}$$

with

8 $$M= \frac{{z}_{1}+{z}_{2}}{{z}_{1}}$$

where ${z}_{1}$ and ${z}_{2}$ represent the distance between the focal point of the illumination beam and sample and that between the sample and detector, respectively. According to the Fresnel scaling theorem, this conical beam geometry can be considered equivalent to a parallel beam configuration, wherein the propagation distance between the sample, detector, and ${{\text{NA}}}_{{\text{det}}}$ is as follows:

9 $${z}_{{\text{eff}}}= \frac{{z}_{2}}{M}$$

and

10 $${{\text{NA}}}_{{\text{det}}}=\frac{m\times \Delta {x}_{{\text{eff}}}}{{2z}_{{\text{eff}}}}.$$

As shown in Fig. 3, the experiment on the USAF resolution test target, wherein ${z}_{2}\hspace{0.17em}$= 46.2 mm and $M\hspace{0.17em}$= 16, resulted in an ${{\text{NA}}}_{{\text{det}}}$ value of 0.14. The improvement in the system design to reduce $z$ or increase $D$ can potentially increase ${{\text{NA}}}_{{\text{det}}}$. Supplementary Figure 1c details the experimental parameters corresponding to each sample measurement.

Characterization of illuminated speckle pattern: ${{\text{NA}}}_{{\text{ill}}}$ of the system induced by the diffuser can be analytically inferred from the intensity pattern obtained using the illumination method^39^. Supplementary Note 2 presents the captured image and a cross-sectional graph of its 2D autocorrelation obtained using weak and strong diffusers. The width of the graph narrowed as the illumination became more diffused, indicating finer patterns. Considering the speckle grain profile generated by the diffuser to be Gaussian, the width of the intensity autocorrelation $w$ is $\sqrt{2}$ times longer than the ensemble average of the speckle grain size $\langle g\rangle$. According to the Fourier relationship, because the incident beam contains spatial high-frequency components, a larger ${{\text{NA}}}_{{\text{ill}}}$ corresponds to a smaller $\langle g\rangle$. Therefore, the relationship can be summarized as follows:

11 $$\langle g\rangle =\frac{w}{\sqrt{2}}=\frac{\lambda }{{{\text{NA}}}_{{\text{ill}}}}.$$

As shown in Supplementary Note 2, the full width at half maximum (FWHM) of the autocorrelation graph was defined in the experiment as $w$. Using a weak and strong diffuser, the measured $w$ was 16.2 and 5.6 μm, respectively. Consequently, the corresponding ${{\text{NA}}}_{{\text{ill}}}$ for the weak and strong diffuser was approximately 0.04 and 0.10, respectively. In the absence of a diffuser, ${{\text{NA}}}_{{\text{ill}}}$ was negligible and assumed to be 0.

The minimum illumination scanning step can be estimated from the autocorrelation graph patterns. For a strong diffuser with a spatial coherence length ${l}_{{\text{c}}}$ of approximately 20 μm, the correlation approaches zero (Supplementary Note 2). Hence, there is an insufficient overlap between adjacent illumination patterns for a scanning step greater than 20 μm. Supplementary Note 9 presents the image reconstruction results while varying the scanning step. Using a strong diffuser and setting the scanning step to 30 μm results in a partially restored image that is blurred at the outer regions. When the scanning step is 40 μm or greater, the image remains unrestored. Conversely, partial image restoration can be achieved with a scanning step of 50 μm using a weak diffuser with a longer coherence length or not using a diffuser at all. Therefore, the minimum scanning step decreases as ${{\text{NA}}}_{{\text{ill}}}$ increases, signifying the selection of appropriate illumination pattern settings to achieve optimal image resolution and measurement time.

${\text{SNR}}$ analysis: ${\text{SNR}}$, an important figure of merit in imaging, is defined as the ratio of the mean intensity of the target image to the standard deviation of the random background noise. To estimate the ${\text{SNR}}$ of an image, the spatial spectrum of the restored object is assumed to be ${\widetilde{O}}_{{\text{r}}}\left(q\right)=F\left\{{O}_{{\text{r}}}(r)\right\}$. The spectral bandwidth of the object ${f}_{w}$ can be expressed as ${f}_{w}={2k}_{0}({{\text{NA}}}_{{\text{det}}}+{{\text{NA}}}_{{\text{ill}}})$, where ${k}_{0}$ represents the magnitude of the wave vector in free space. Here, ${N}_{{\text{o}}}$ is defined as the number of orthogonal modes within the spectral range, implying that $\sqrt{{N}_{{\text{o}}}}$ is proportional to ${{\text{NA}}}_{{\text{ill}}}$. The signal ${I}_{{\text{sig}}}$, denoting the intensity of the reconstructed target, is given as ${I}_{{\text{sig}}}={\alpha N}_{{\text{o}}}$, where $\alpha$ corresponds to the Strehl ratio after image reconstruction. Owing to the stochasticity of noise, the standard deviation of the measurement noise ${\upsigma }_{{\text{back}}}$ after image reconstruction remains nearly constant and proportional to the square root of ${N}_{{\text{o}}}$, i.e., ${\upsigma }_{{\text{back}}}=\beta \sqrt{{N}_{{\text{o}}}}$, where $\beta$ represents the measurement noise in a single wide-field image^40^. This measurement noise includes the reconstruction error, photon shot noise, and dark count noise originating from the camera sensor. Because ${\text{SNR}}$ is determined by the ratio of the reflected signal to background fluctuation, the ${\text{SNR}}$ of the speckle illumination is proportional to $\sqrt{{N}_{{\text{o}}}}$, i.e., ${{\text{NA}}}_{{\text{ill}}}$. In the proof-of-concept simulation presented in Fig. 4, the ${\text{SNR}}$ increased proportionally with ${{\text{NA}}}_{{\text{ill}}}$, corresponding to the theoretical expectation.

### Supplementary Information

Supplementary Information 1.

Supplementary Video 1.

## Data Availability

The datasets generated in this study are available from the corresponding author upon request.

## Code availability

The code is available from the corresponding author upon reasonable request.
