# Supplementary material for: Lens-free reflective topography for high-resolution wafer inspection
Source: Sci Rep. 2024 May 8;14:10519. doi: 10.1038/s41598-024-59496-4 (PMC11076508; doi:10.1038/s41598-024-59496-4)
Supplement: Supplementary file 1 — Supplementary Information 1. [file 41598_2024_59496_MOESM1_ESM.docx]

**Supplementary Information**

Lens-free reflective topography for super-resolution wafer inspection

Hojun Lee^1*^, Jangwoon Sung^1^, Seungbeom Park^1^, Junho Shin^1^, Hyungjin Kim^1^, Wookrae Kim^1^

and Myungjun Lee^1^

^1^*Mechatronics Research, Samsung Electronics Co., Ltd., 1-1 Samsungjeonja-ro, Hwaseong-si, Gyeonggi-do, 18848, Korea*

[^*^*hojun86.lee@samsung.com*](mailto:*hojun86.lee@samsung.com)

# Supplementary Note 1: Detailed experimental system

*
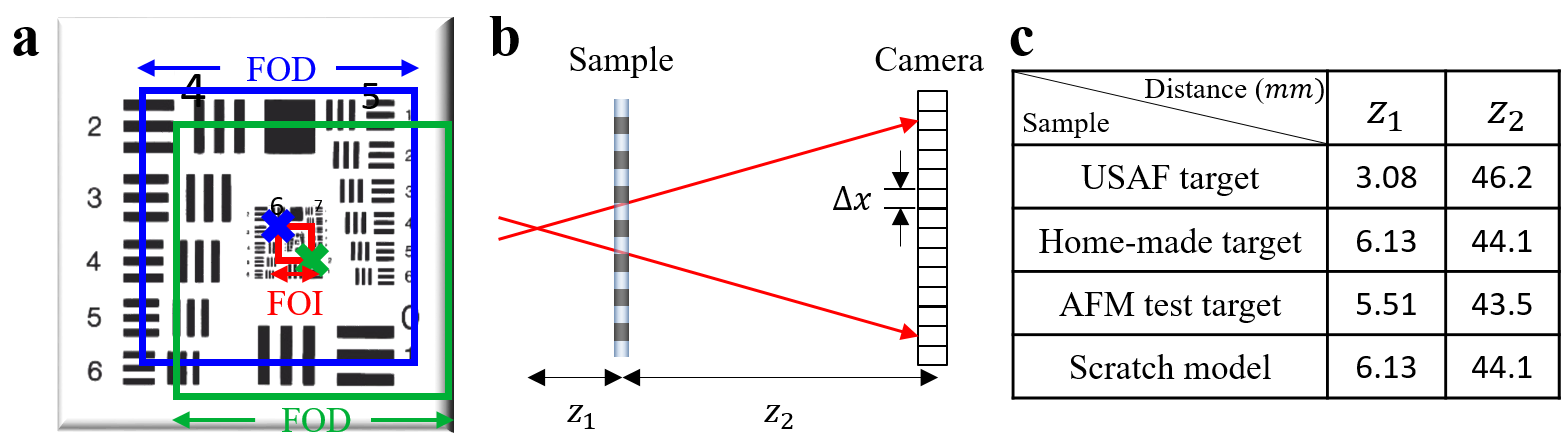
*

**Supplementary Figure 1. Details of the experimental setup. a.** Diagram showing the FOI and FOD in the measurement of the USAF resolution test target. **b.** Transmissive representation of the Re-SLIM system for visual clarity. **c.** The values of $z_{1}$ and $z_{2}$ for each experiment.

For all experiments, the FOI, indicated by the red box in Supplementary Fig. 1a, was fixed at 100 $\times$ 100 $\mu$m^2^. The blue and green boxes represent the FOD values for the two representative illumination points, indicated by the blue and green $\times$ marks, respectively. The FOD for the USAF resolution test target was set to 830 $\times$ 830 $\mu$m^2^. The FOI and FOD are determined by the scanning range and the number of used CCD pixels, respectively. Therefore, the FOI and FOD can be independently controlled in a Re-SLIM system based on the desired FOV.

In Supplementary Fig. 1b, $z_{1}$ denotes the distance between the focal point of the incidence beam and the sample and $z_{2}$ denotes that between the sample and CCD. Owing to beam divergence characteristics, the sample plane is magnified and captured by the detector plane with a magnification factor $M$ described by Eq. (8) in the main text. The actual size of a single CCD pixel is represented as $\Delta x$. The effective pixel size $\Delta x_{\mathrm{eff}}$ and sample-to-detector distance $z_{\mathrm{eff}}$ are determined using Eqs. (7) and (9), respectively. Consequently, $\mathrm{NA}_{\det}$ is calculated using Eq. (10). Supplementary Fig. 1c presents the values of $z_{1}$ and $z_{2}$ for each experiment.

# Supplementary Note 2: Analysis of the illuminated speckle pattern characteristics

*
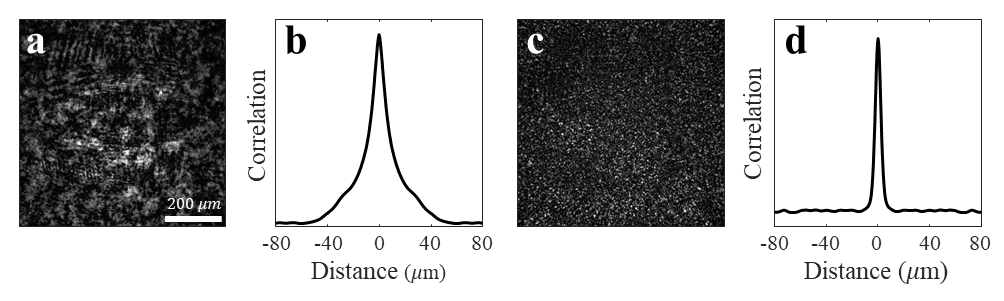
*

**Supplementary Figure 2. Speckle pattern analysis for various diffusers.** Measured raw images of the USAF resolution test target with **a.** strong and **b.** weak diffusers, respectively. **b.** and **d.** Cross-sectional plots of 2D autocorrelation for intensity images in **a** and **c**, respectively. The FWHM values for **b** and **d** are 16.2 and 5.6 $\mu$m, respectively.

In optical imaging, the speckle effect is observed during the interaction of coherent light, such as a laser, with a rough or diffusive surface. The mean speckle size, a crucial parameter in speckle pattern analysis, depends on the properties of the random surface or medium. This parameter is important in practical applications, such as surface roughness measurement, optical thickness determination, and particle size estimation in scattering media. We aim to predict the diffuser-induced $\mathrm{NA}_{\mathrm{ill}}$ of the system using the speckle pattern determined by the illumination method.

The speckle pattern appears as a random arrangement of bright and dark spots resulting from the interference of multiple plane waves. Consider the following speckle electrical-field:

| $\boldsymbol{E}_{s}=\sum_{j=1}^{N_{i}} k_{j}\boldsymbol{q}_{j}$, | (S1) |
| --- | --- |

where $\boldsymbol{q}_{j}$ represents the wavevector of the j-th parallel wave and $k_{j}$ denotes the random coefficient. The number of orthogonal modes $N_{i}$ required to represent the speckle pattern increases with $\mathrm{NA}_{\mathrm{ill}}$. When the object is mirror-like compared to the speckle pattern, the measured intensity image can be expressed as $I=\left| \boldsymbol{E}_{s} \right|^{2}$ and the 1D intensity autocorrelation function is given as follows:

| $A\left( \delta\right)=\int I\left( x \right)I\left( x-\delta\right)dx$, | (S2) |
| --- | --- |

where $\delta$ represents the displacement in the $x$-direction. The integral signifies that the mean speckle size of the entire detected area is calculated as an average of the profile speckle sizes. The mean speckle size $\left\langle g \right\rangle$, which corresponds to the width of the $A(\delta)$, can be used to estimate the $\mathrm{NA}_{\mathrm{ill}}$ value of the system. Supplementary Fig. 2 shows instances of raw images and their corresponding cross-sections for 2D autocorrelation functions. The width of the autocorrelation function $w$ is defined as the FWHM. Assuming that the speckle grain profile generated by the diffuser follows a Gaussian distribution, $w$ is approximately $\sqrt{2}$ times longer than $\left\langle g \right\rangle$. From Eq. (S1), $\mathrm{NA}_{\mathrm{ill}}$ depends on the effectiveness of the diffuser in generating smaller speckle patterns, with a larger $N_{i}$ contributing to the overall formation of the speckle pattern. The Fourier relationship between the speckle image and $\mathrm{NA}_{\mathrm{ill}}$, described in Eq. (11), indicates that $\mathrm{NA}_{\mathrm{ill}}$ can be predicted as $\mathrm{NA}_{\mathrm{ill}}=\lambda\frac{\sqrt{2}}{w}$. Therefore, the expected values of $\mathrm{NA}_{\mathrm{ill}}$ owing to weak and strong diffusers are estimated as 0.04 and 0.10, respectively.

# Supplementary Note 3: FOV expansion by the speckle illumination effect


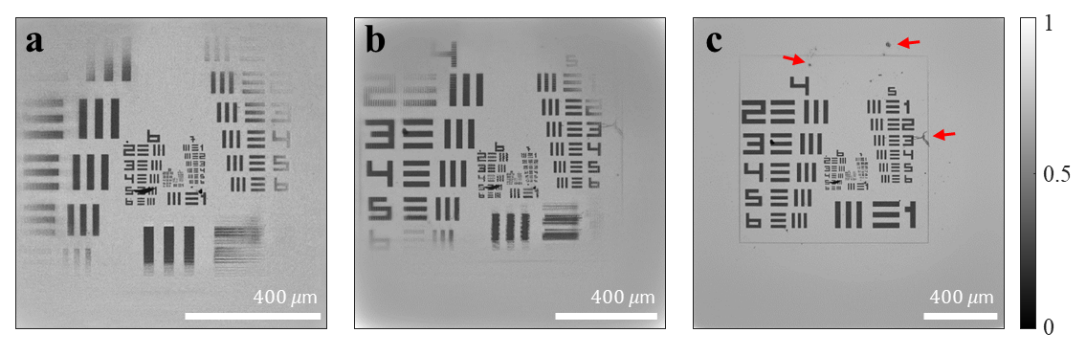


**Supplementary Figure 3. FOV increase based on the image reconstruction pixel count. a.** Image reconstructed with the original sensor pixel count (2048 $\times$ 2048 pixels) using a strong diffuser. Including the FOI, the total number of pixels representing this image is 2303 $\times$ 2303 corresponding to an FOV of 0.9 $\times$ 0.9 mm^2^. Images restored with pixel padding, resulting in a total of **b.** 3108 $\times$ 3108 and **c.** 4325 $\times$ 4325 pixels corresponding to FOVs of 1.3 $\times$ 1.3 and 1.8 $\times$ 1.8 mm^2^, respectively. For FOV comparison, the scale bar is fixed at 40 $\mu$m. In **c**, the imaging captures the glass edge of the USAF resolution test target and additional debris (indicated by red arrows).

Owing to its diffusivity, the speckle illumination effect expands the illuminated area. Beyond the FOD, the sensor captures scattered signals from the surroundings, providing computational advantages for broadening the FOV in image restoration. This phenomenon originates from the near-field measurements. Analogous to the increase in spectral bandwidth within the spatial spectrum domain caused by speckle illumination effects, the imaging capabilities in real space are extended beyond the FOD (Figs. 3a, e, and i of the main text). However, if the pixel count in the real space is insufficient, it becomes necessary to increase the pixel count through padding in the image restoration process to represent a broader FOV. In the USAF resolution test target imaging using a strong diffuser with a fixed sensor pixel count (2048 $\times$ 2048 pixels) and iterative algorithm, the probe function is restored only up to the FOD size. Consequently, the FOV is limited to 0.9 $\times$ 0.9 mm^2^ in the restored image (Supplementary Fig. 3a). For image representation, increasing the total pixel count to 3108 $\times$ 3108 or 4325 $\times$ 4325 pixels using pixel padding results in an expanded FOV, as shown in Supplementary Figs. 3b and c with FOVs of 1.3 $\times$ 1.3 and 1.8 $\times$ 1.8 mm^2^, respectively. Supplementary Fig. 3c suggests that imaging is possible even for the target features, such as glass edge shapes and debris, indicated by the red arrows. However, an increase in pixel count, which denotes the volume of information processed during image restoration, increases computation times. The restoration times in Supplementary Figs. 3a, b, and c were 335, 818, and 1,033 s, respectively. Therefore, the targeted FOV and throughput should be optimally balanced.

# Supplementary Note 4: Determination of the TV regularization coefficient


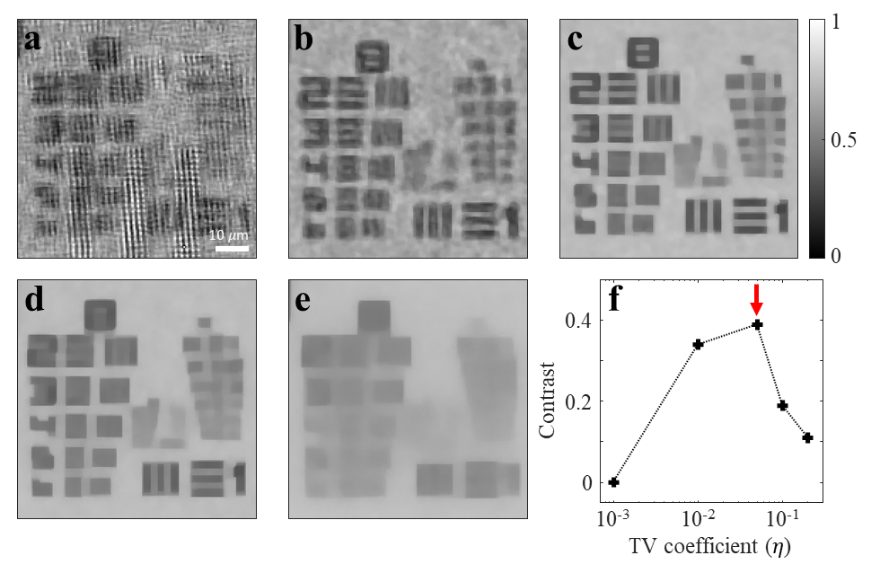


**Supplementary Figure 4. Comparison of restored images based on the TV regularization coefficient. a–e.** Converged images corresponding to the TV regularization factor $\eta$ values of 0.001, 0.01, 0.05, 0.1, and 0.2 while using a strong diffuser. In **f**, the optimal $\eta$ value is approximately 0.05 based on the contrast comparison graph of the restored images.

TV regularization is a commonly employed noise reduction technique in digital image processing. Signals with excessive detail have high TV values; hence, the TV regularization technique removes unnecessary noise while preserving essential details, such as structural edges. If the TV regularization coefficient $\eta$ is too small, the noise filtering effect becomes negligible; otherwise, it affects the signal owing to the fine structure of objects. Additionally, $\eta$ affects the convergence speed of the image, making its selection dependent on the imaging target.

The optimal value of $\eta$ can be systematically calculated by monitoring the contrast of the recovered image. The image restoration algorithm is designed to deconvolve the probe $\psi$ and object $O$ functions. The contrast of the reconstructed image increases when the algorithm performs effectively. To validate this, we selected a 71 $\times$ 71 $\mu$m^2^ area from the USAF resolution test target image restored using a strong diffuser. The image restoration algorithm was applied to this area while gradually adjusting $\eta$ (Supplementary Figs. 4a–e), and the contrast of the reconstructed image was plotted as a function of $\eta$ as shown in Supplementary Fig. 4f. The contrast was maximum at $\eta=0.05$ (indicated by the red arrow), where the fine structures of the target are most distinct.

# Supplementary Note 5: Enhancing throughput using the image optimization algorithm

*
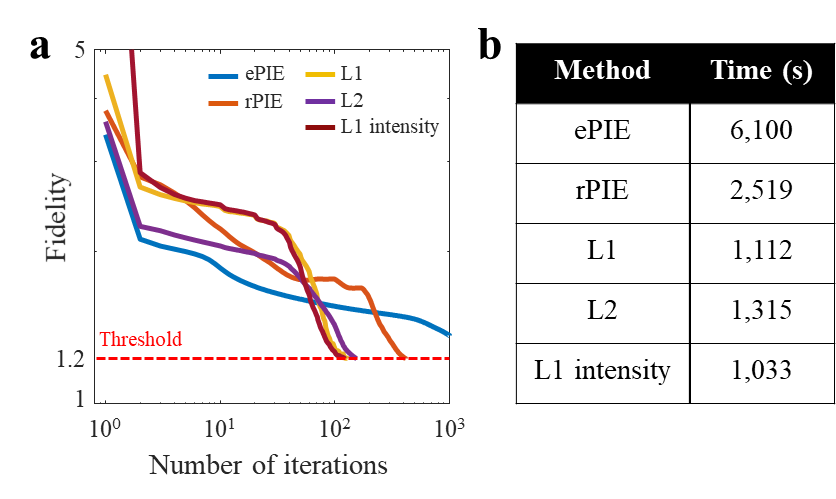
*

**Supplementary Figure 5. Comparison of the computational speeds of various image reconstruction algorithms. a.** Graph showing decrease in fidelity with respect to the number of iterations for each algorithm. The calculation converges to the final image when the fidelity attains a threshold. **b.** Convergence time for each algorithm, indicating the instant at which the fidelity attains the threshold.

The final image reconstruction time varies depending on the employed cost function, including the conventional ptychographic algorithm, which impacts the imaging throughput. Therefore, the selected algorithm should be appropriate for imaging the target of interest. We compared the convergence speeds of various image reconstruction algorithms for the USAF resolution test target shown in Fig. 3 of the main text. Supplementary Figure 5a presents a graph showing the decrease in image fidelity with the number of iterations. Fidelity $F$ is defined as

| $F=\log_{10} \left\Vert\sqrt{I}-\sqrt{I_{r}} \right\Vert_{2}^{2}$. | (S3) |
| --- | --- |

We assumed that attaining a pre-determined fidelity value (indicated by red dashed line) corresponds to the convergence of the final objective $O_{r}$ and probe $\psi_{r}$ functions. Supplementary Figure 5b provides a summary of the final convergence times converted into time units. To ensure a fair comparison of the final convergence time, the FOI and FOD were fixed at 100$\times$100 and 830 $\times$ 830 $\mu$m^2^, respectively. The images comprising 3768 $\times$ 3768 pixels were processed using the same computational resources (NVIDIA A100 40 GB) for all algorithms. For targets with sharp pattern edges and high contrast, such as the USAF resolution test target, the algorithms employing the L1 norm applied to intensity image (‘L1 intensity’ in Supplementary Figure 5) as the cost function demonstrated a 5.9- and 2.4-fold faster convergence compared with the widely-used ptychographic algorithms ePIE and rPIE, respectively. The absolute speed could further improve depending on the FOI, FOD, and number of image measurements. Reference 30 in main text provides detailed information regarding ePIE, rPIE, and the cost function used in each algorithm.

# Supplementary Note 6: Details of image reconstruction algorithm


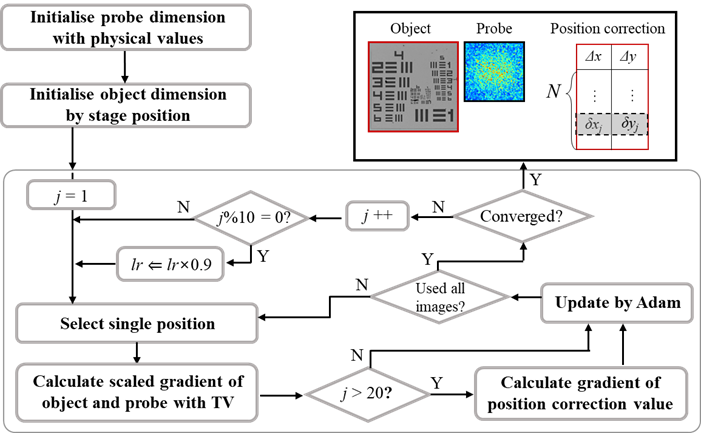


**Supplementary Figure 6. Strategic flowchart for rapid and accurate convergence of image and probe functions.** The changes in the object and probe functions updated at each iteration can be controlled by the learning rate (*lr*). If the *lr* is small from the early iterations, it leads to slower convergence as it requires more iterations to reconstruct the image. Conversely, if the *lr* is large in the later iterations, there is a wider swing around the global minimum point of the loss function (Eq. 3 in main text), making it difficult to reconstruct the accurate image. Therefore, we provide an adaptive *lr* value that changes with every ten iterations to address these issues. Furthermore, by updating the position error *(*$\Delta x$,$\Delta y$) after a certain number of iterations (*j* > 20), we prevent the divergence of the functions in the early stages.

The proposed reconstruction algorithm shown in Supplementary Figure 6 uses the Adam optimizer to determine the optimal complex profiles of $O_{r}$ and $\psi_{r}$, and selectively for the stage position error correction. An initial learning rate (*lr*) of 10^-2^ was applied to the probe and image. To suppress the rapid variation in the image position, the learning rate was scaled to 10^-5^ for the stage position error correction. Similarly, the stage error was updated after the 20^th^ iteration. The learning rate was reduced by 10% for every ten iterations, thus minimizing the elapsed time. Other hyperparameters, such as *β*_1_ and *β*_2_ with values 0.9 and 0.999, respectively, remained constant without using the weight decay. A unit batch size was selected because using a greater value decreased the resultant image quality and convergence speed.

We introduced various strategies for accelerating the convergence time. Reconstruction using speckle illumination often produces the incorrect outcome, such as distortion or partial absence. Such outcomes are attributed to the initial inaccuracy of stage position, complexity of probe profile, and corresponding rearrangement of the original sample image information in the captured data. These factors are related to the computational image reconstruction problem, which is observed for the measured samples with periodic structures. This issue can be solved by performing additional iterations of the ptychographic algorithm, thanks to its self-calibration effect. However, the Adam optimizer worsens this effect because it exponentially accumulates the gradient momentum. After satisfying a criterion for sufficiently low loss value, pulling the optimized values out of local minimum becomes difficult. To address this problem, the gradient values applied to $O_{r}$ and $\psi_{r}$ are always scaled to a constant and identical value. Hence, instead of relying on their gradient weakening owing to continuously reducing loss value, this strategy makes the update equivalently robust throughout optimization and effective to the spatial distributions of $O_{r}$ and $\psi_{r}$. As a result, the total convergence time decreases, and the sharpening effect of total variation is suppressed, since its contribution always become lower than that from loss. Pytorch was implemented for all computational procedures.

# Supplementary Note 7: Scanning position error correction for high-resolution image reconstruction

*
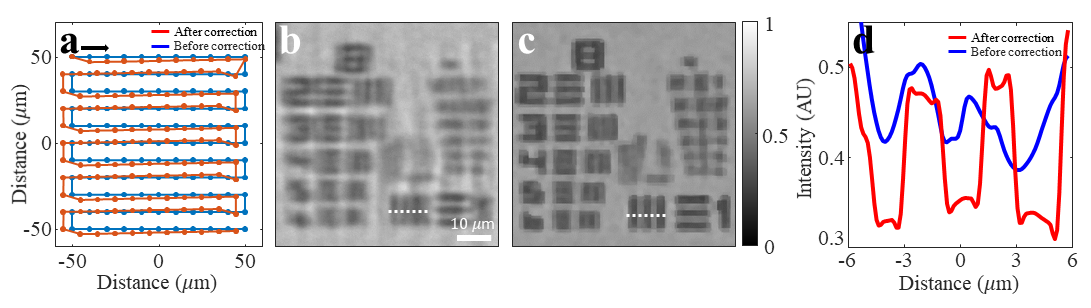
*

**Supplementary Figure 7. Comparison of position error correction before and after. a.** Scanning positions before (blue line) and after (red line) error correction. Reconstructed images **b.** before and **c.** after scanning position error correction. **d.** Line plot graphs of regions indicated by the white dashed lines in **b** and **c**.

Re-SLIM requires image scanning similar to other computational imaging techniques. The scan position should be accurately fed into the algorithm owing to the discrepancy between the measured and actual illumination positions in experimental settings. The final image quality is influenced by the ability to compensate for this positional error $(\delta x_{j}$, $\delta y_{j})$. To rectify these errors, an additional layer was introduced into the optimization algorithm used in Re-SLIM. Supplementary Fig. 7a shows the raster scan position before and after error correction. The variation in scanning direction along the horizontal axis introduces an error of approximately a few micrometers, resulting in significant errors owing to sample stage backlash. Supplementary Fig. 7b shows that the absence of compensation distorts the image; thus, the expected resolution is not achieved. However, the expected resolution is achieved in the restored image with corrected scan position errors, as shown in Supplementary Fig. 7c. This can be inferred from the cross-sectional plot in Supplementary Fig. 7d.

# Supplementary Note 8: Image analysis in spatial spectrum domain

*
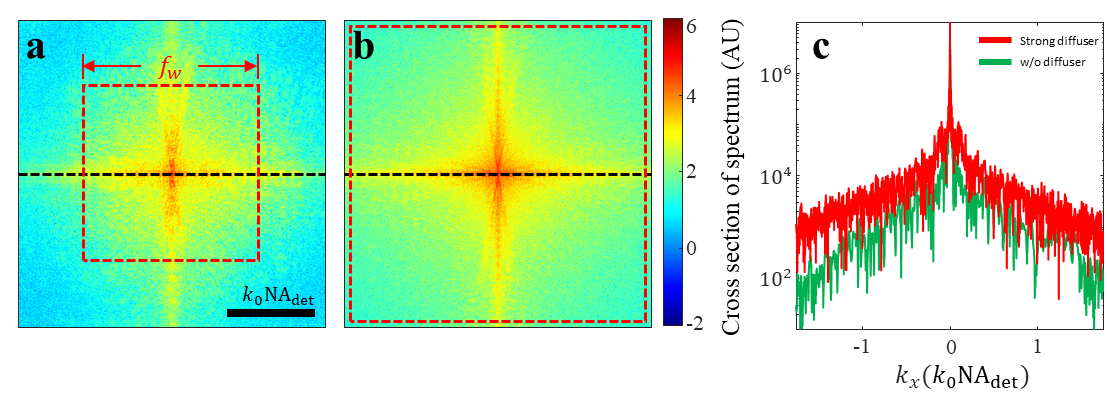
*

**Supplementary Figure 8. Spatial spectrum images with and without speckle illumination effects.** The 2D Fourier-transformed images of **a.** Fig. 3a and **b.** Fig. 3i in the main text. The red dashed box represents the spectral bandwidth $f_{w}$. Owing to the diffuser-induced increase in $\mathrm{NA}_{\mathrm{ill}}$, the value of $f_{w}$ in **b** increases compared to that in **a**. **c.** Cross-section graphs of the black dashed lines in **a** and **b**, with the y-axis on a logarithmic scale.

In general optical imaging, the number of orthogonal modes $N_{o}$ required to represent an image is given by

| $N_{o}=\left( \frac{\sqrt{\mathrm{FOV}}}{d} \right)^{2}$, | (S4) |
| --- | --- |
|  |  |

which is the minimum degrees of freedom required to represent a specific FOV image with a resolution of $d$. The value of $N_{o}$ remains constant in the spatial spectrum domain. All $N_{o}$ orthogonal modes are included within the spectral range $f_{w}={2k}_{0}(\mathrm{NA}_{\det}+\mathrm{NA}_{\mathrm{ill}})$, where $k_{0}=\frac{2\pi}{\lambda}$, covered by the overall $\mathrm{NA}$ of the system. Based on Eq. (10), $\mathrm{NA}_{\mathrm{ill}}$ is negligible when $\mathrm{NA}_{\det}$ is constant at 0.14, as in the case of Supplementary Fig. 8a. However, as shown in Supplementary Fig. 8b, Eq. (11) yields the diffuser-induced $\mathrm{NA}_{\mathrm{ill}}$ value of 0.10. Based on the cross-sectional graph in Supplementary Fig. 8c, the diffuser-induced increase in $\mathrm{NA}_{\mathrm{ill}}$ results in an expanded spectral width.

By substituting Eq. (1) into Eq. (S4), $\mathrm{NA}$ is directly proportional to $\sqrt{N_{o}}$, which can be expressed as $f_{w}\propto\sqrt{N_{o}}$ in the spatial spectral domain. Based on the statistics of the sum of real variables, the reflective signal $I_{\mathrm{sig}}$ from the target that remains constant during measurements is proportional to $N_{o}$, whereas the randomly varying noise $\sigma_{\mathrm{back}}$ increases with $\sqrt{N_{o}}$. Consequently, the $\mathrm{SNR}=\frac{I_{\mathrm{sig}}}{\sigma_{\mathrm{back}}}$ is proportional to $\sqrt{N_{o}}$ or $\mathrm{NA}_{\mathrm{ill}}$, as described in the main text.

# Supplementary Note 9: Systematic search for the optimal scanning steps

*
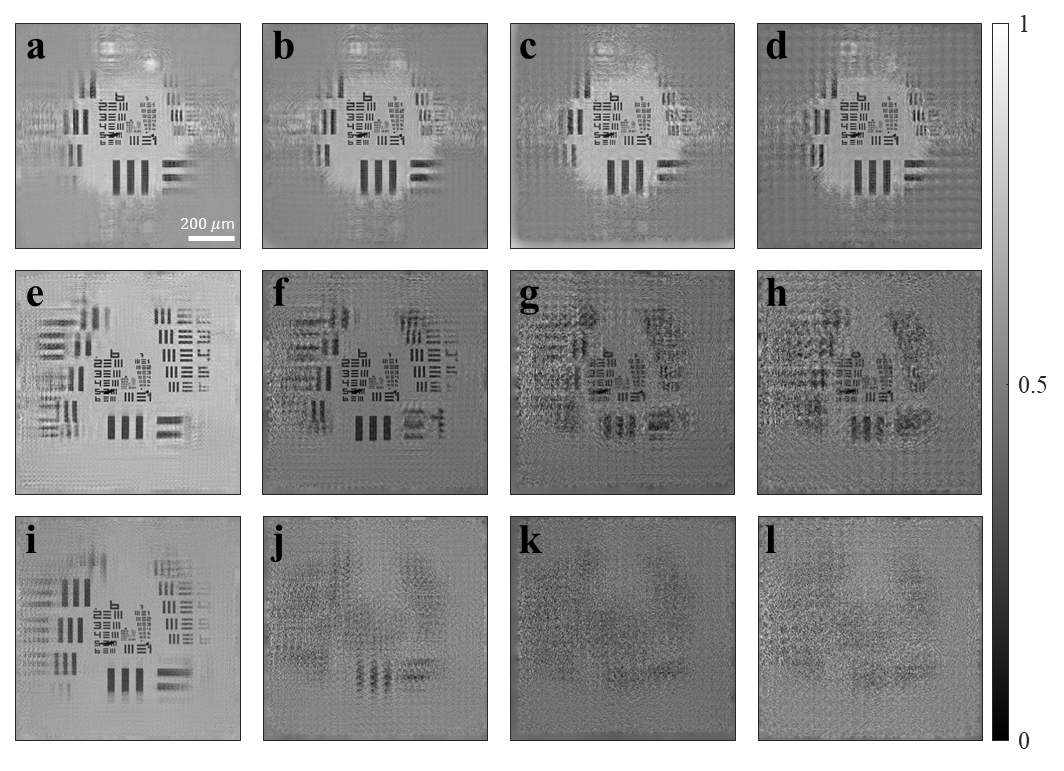
*

**Supplementary Figure 9. Image reconstruction results at various scanning steps. a–d.** Reconstructed images obtained using divergence wave illumination at scanning steps of 20, 30, 40, and 50 $\mu$m, respectively. Reconstructed images obtained using **e–h.** a weak diffuser and **i–l.** a strong diffuser, with scanning steps arranged in the order **a–d**. All images are represented using 2355 $\times$ 2355 pixels.

The image scanning technique used in Re-SLIM relies on the scanning step, which is a crucial factor. The overlap of optical information detected between neighboring illuminations is essential for image restoration algorithms. Because the scanning step depends on illumination patterns, determining its appropriate value corresponding to each pattern is crucial. Hence, we experimentally and analytically investigated the optimal scanning steps for various illumination patterns. As shown in Supplementary Figs. 9a–d, the image could be restored for the cone-shaped wave illumination using a maximum scanning step of 50 $\mu$m. However, for speckle pattern illumination in Supplementary Figs. 9e–h, a scanning step of approximately 30 $\mu$m was required. As shown in Supplementary Figure 9i-l, a minimum scanning step of 30 $\mu$m was insufficient for proper image restoration using a strong diffuser. The optimal scanning step for each illumination pattern can be determined by analyzing the illumination patterns. Herein, the spatial coherence length $l_{c}$ satisfying $A\left( l_{c} \right)=0$ in Eq. (S2) serves as a reference point, indicating the distance between adjacent illuminations without correlation. For a scanning step $\Delta s$ greater than $l_{c}$, there is insufficient overlap of information between the measured diffraction images; hence, the image cannot be restored. Conversely, $\Delta s$ < $l_{c}$ results in reduced throughput for practical applications. Consequently, $\Delta s$ $\approx$ $l_{c}$ is the optimal scanning step for reconstructing the final image from the overlapped signals at each scanning position. Using a strong diffuser, the experimentally calculated value of $l_{c}$, i.e., 20 $\mu$m, is in accordance with the minimum scanning step determined for image restoration.
